# Blunt Chest Trauma Causing a Displaced Sternal Fracture and ST-elevation Myocardial Infarction: A Case Report

**DOI:** 10.5811/cpcem.2020.12.49875

**Published:** 2021-01-19

**Authors:** Keaton Nasser, Jaclyn Matsuura, Jimmy Diep

**Affiliations:** * University of Nevada Las Vegas, Department of Cardiology, Las Vegas, Nevada; † University of Nevada Las Vegas, Department of Emergency Medicine, Las Vegas, Nevada; ‡ Nevada Heart and Vascular Center, Department of Cardiology, Las Vegas, Nevada

**Keywords:** STEMI, blunt chest trauma, sternal fracture

## Abstract

**Introduction:**

Blunt chest trauma and motor vehicle collisions are common presentations to the emergency department (ED). Chest pain in a trauma patient can usually and reasonably be attributed to chest wall injury, leading to a potential delay in diagnosis and treatment.

**Case Report:**

In this case report, we present a 52-year-old male who was brought to the ED with complaints of chest pain and pressure after a motor vehicle collision. He was subsequently found to have both a displaced sternal fracture and simultaneous acute myocardial infarction with 100% occlusion of the mid left anterior descending artery without dissection requiring stent placement.

**Conclusion:**

Chest pain after blunt cardiac trauma is a common complaint. While rare, acute myocardial infarction must be considered. Most injuries result as direct trauma to the artery causing either dissection or acute thrombosis resulting in a myocardial infarction as opposed to acute plaque rupture with thrombosis, as seen in this case.

## INTRODUCTION

Blunt chest trauma can cause a variety of cardiac injuries. Chest pain in a trauma patient can usually and reasonably be attributed to chest wall injury, leading to a potential delay in diagnosis and treatment. Myocardial infarction from artery dissection, thrombosis from trauma, and plaque rupture need to be considered in the differential. The incidence of coronary artery injury in blunt chest trauma is approximately 2%, with most reported cases being coronary artery dissections.^1^ Blunt cardiac injury has been reported as the most overlooked injury in patients who die from trauma; therefore, emergency providers should maintain a high index of suspicion.^2^ We report on a patient with a displaced sternal fracture and simultaneous acute myocardial infarction as a result of blunt chest trauma.

## CASE REPORT

**Image 1:**
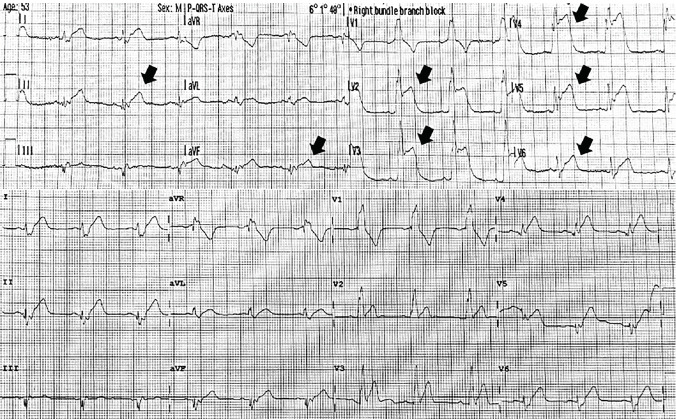
Electrocardiograms obtained at the collision scene (top) showing ST elevations (arrows) in leads V2–V6 and leads II and aVF; and on hospital arrival (bottom) showing sinus rhythm with right bundle branch block.

A 52-year-old male was brought to the emergency department (ED) via emergency medical services (EMS) with complaints of chest pain and pressure after a motor vehicle collision. The patient was the restrained driver in a collision while traveling at approximately 45 miles per hour when a car turned in front of him at an intersection. His airbags deployed; he did not lose consciousness and was able to self-extricate from the vehicle. After extrication, he started to experience substernal chest pressure, which was not present prior to the accident. When EMS arrived he was placed in a cervical collar and given aspirin, and an electrocardiogram (ECG) was obtained (Image 1). He was transported to the ED.

On arrival, a repeat ECG was performed, and the emergency physician alerted a cardiac activation given concern for an ST-elevation myocardial infarction (STEMI). He complained of substernal chest pain and pressure that was non-radiating and exacerbated by deep inspiration. Additional review of systems was negative.

On examination in the ED, the patient’s temperature was 36.6° Celsius, heart rate 76 beats per minute, respirations 15 breaths per minute, blood pressure 102/60 millimeters mercury, and an oxygen saturation of 97%. He was in no acute distress. He had tenderness to palpation over the sternum with no evidence of contusion or seatbelt sign on chest or abdomen. He had no abnormalities on cardiac, lung, or abdominal exams. He had no bony point tenderness of extremities and had full range of motion of all extremities.

He had no prior medical history but had not had regular medical care in the prior 30 years. He had smoked 1.5 packs per day of cigarettes for many years, consumed alcohol on social occasions, and denied recreational drug use. He had no known drug allergies. He had never had surgery. He denied any family history of coronary artery disease and denied any personal history of hypertension, hyperlipidemia, or coronary artery disease.

Chest radiography demonstrated only mild left lung base atelectasis. Point-of-care echocardiography by the cardiology service was limited by difficult windows and pain on transducer pressure, but demonstrated a relatively preserved ejection fraction, no pericardial effusion, and no obvious wall motion abnormalities. Initial troponin-I was elevated at 0.045 nanograms per milliliter (ng/mL) (reference range 0.02–0.04 ng/mL), and B-type natriuretic peptide was 10 picograms (pg)/mL (<=99 pg/mL).

**CPC-EM Capsule**

What do we already know about this clinical entity?

Blunt chest trauma and motor vehicle collisions are common presentations in the emergency department. Chest pain is a common complaint.

What makes this presentation of disease reportable?

We report the simultaneous presentation of a displaced sternal fracture and acute myocardial infarction in the setting of blunt chest trauma.

What is the major learning point?

Blunt cardiac injury is the most commonly overlooked injury in patients who die from trauma. Acute myocardial infarction and cardiac injury must be considered.

How might this improve emergency medicine practice?

Suspicion should be high for cardiac injury, even in the setting of reproducible “musculoskeletal” chest pain.

**Image 2:**
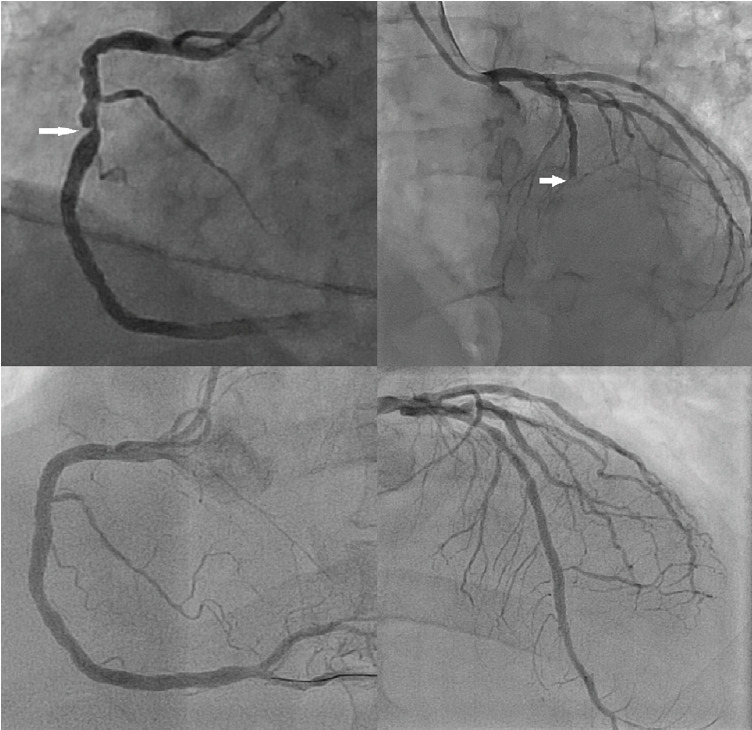
Coronary angiography showing 100% occlusion of the mid-left anterior descending artery (right) and 70–90% stenosis of the right coronary artery (left) in the upper images, and the subsequent corresponding angiographic results after stenting in the lower images.

**Image 3:**
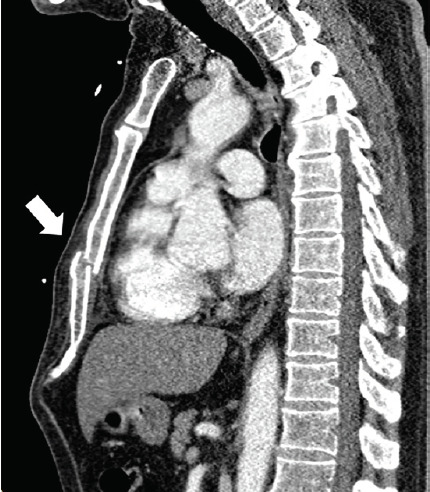
Chest computed tomography showing a displaced sternal fracture (arrow).

While the diagnosis of cardiac contusion was entertained, the field ECG could not be ignored. With no contraindications to angiography identified, the patient was fully anticoagulated and taken emergently for coronary angiography. Coronary angiogram demonstration 100% occlusion of the mid left anterior descending artery (LAD) without dissection (Image 2). There was also a mid-right coronary artery (RCA) ragged 70–90% lesion. After intervention on the LAD lesion, the patient’s pain subsided. The next day, he complained of chest pain on deep inspiration and chest heaviness. He had a staged intervention of the RCA the following day. In the interim, he was discovered to have a sternal fracture by computed tomography (Image 3).

## DISCUSSION

The patient’s field ECG showed sinus rhythm, a right bundle branch block, and ST-segment elevations in leads V2–V6, consistent with acute anterior injury, and leads II and aVF consistent with acute inferior injury. The ECG obtained in the ED demonstrated sinus rhythm and a right bundle branch block. However, the previous ST-segment elevations were no longer as profound and there were no developing q-waves consistent with the natural progression of ECG changes in ST-elevation myocardial infarctions (STEMI). The degree of variance between these two ECGs could have been simply explained by lead position. The widespread ST changes in multiple territories compounded the diagnosis. Cardiac contusion can have any ECG findings, including ST elevations.^3^ The case was further complicated by the patient’s significant trauma and reproducible chest pain, which could have been musculoskeletal.

Blunt thoracic trauma can cause a multitude of cardiac injuries. They range from benign and clinically silent cardiac contusion to cardiac wall rupture. Pericardial injuries, valvular injuries, and coronary artery injuries are also possible. Providers should be wary of attributing ECG changes to cardiac contusion alone.

A PubMed search using the terms “STEMI” and “blunt chest trauma” revealed fewer than 30 full cases published in English in the prior 20 years. The cause of STEMI was dissection in 13 patients, acute plaque rupture or thrombosis in 9 patients, cardiac contusion in 1 patient, and an artery to ventricle fistula in 1 patient. Only our case showed ECG changes in more than one territory. Coronary artery occlusions from trauma are a relatively rare phenomenon with three possible mechanisms. Direct impact may cause intimal disruption and dissection. Alternatively, impact can cause acute intra-arterial thrombosis with or without the involvement of acute plaque rupture involvement (ie, thrombosis can occur in normal coronary arteries without other evidence of native atherosclerotic disease). All three types can result in a STEMI.

Treatment can include emergency coronary bypass surgery, percutaneous stenting, aspirational thrombectomy without stenting, or medical management. Invasive angiography is necessary for diagnosis and to determine the treatment course. These injuries can happen in individuals of any age and with any level of intimal plaque.^4^–^9^ Because of its anterior location and its position lying behind the sternum, the left anterior descending artery tends to be the culprit. However, abdominal injuries with an upward force can affect the inferior vessels. There are at least two reports of circumflex artery involvement, but both of those cases also involved the LAD.^10^,^11^ The mechanism for left circumflex artery injury is unclear.

## CONCLUSION

Chest pain after blunt cardiac trauma is a common complaint, and while rare, acute myocardial infarction must be entertained. Blunt cardiac injury has been cited as the most commonly overlooked injury in patients who die from trauma.^2^ Most injuries result as a direct trauma to the artery causing either dissection or acute thrombosis resulting in a myocardial infarction as opposed to acute plaque rupture with thrombosis, as seen in this case. Our patient was fortunate to not have injuries that would preclude invasive angiography and percutaneous intervention. Trauma patients with coexisting injuries may not be candidates for fibrinolytic therapy because of the risk of hemorrhage. Given the emergent need for coronary angiography, emergency providers should maintain a high index of suspicion for STEMI in the setting of blunt chest trauma.
